# Requirement or exclusion of inverted repeat sequences with cruciform-forming potential in *Escherichia coli* revealed by genome-wide analyses

**DOI:** 10.1007/s00294-018-0815-y

**Published:** 2018-02-27

**Authors:** Osamu Miura, Toshihiro Ogake, Takashi Ohyama

**Affiliations:** 1 Department of Biology, Faculty of Education and Integrated Arts and Sciences, Waseda University, 2-2 Wakamatsu-cho, Shinjuku-ku, Tokyo, 162-8480 Japan; 2 Major in Integrative Bioscience and Biomedical Engineering, Graduate School of Science and Engineering, Waseda University, 2-2 Wakamatsu-cho, Shinjuku-ku, Tokyo, 162-8480 Japan

**Keywords:** Cruciform, *E. coli*, Genome-wide distribution, Intrinsic terminator, Inverted repeat (IR) sequence

## Abstract

Inverted repeat (IR) sequences are DNA sequences that read the same from 5′ to 3′ in each strand. Some IRs can form cruciforms under the stress of negative supercoiling, and these IRs are widely found in genomes. However, their biological significance remains unclear. The aim of the current study is to explore this issue further. We constructed the first *Escherichia coli* genome-wide comprehensive map of IRs with cruciform-forming potential. Based on the map, we performed detailed and quantitative analyses. Here, we report that IRs with cruciform-forming potential are statistically enriched in the following five regions: the adjacent regions downstream of the stop codon-coding sites (referred to as the stop codons), on and around the positions corresponding to mRNA ends (referred to as the gene ends), ~ 20 to ~45 bp upstream of the start codon-coding sites (referred to as the start codons) within the 5′-UTR (untranslated region), ~ 25 to ~ 60 bp downstream of the start codons, and promoter regions. For the adjacent regions downstream of the stop codons and on and around the gene ends, most of the IRs with a repeat unit length of ≥ 8 bp and a spacer size of ≤ 8 bp were parts of the intrinsic terminators, regardless of the location, and presumably used for Rho-independent transcription termination. In contrast, fewer IRs were present in the small region preceding the start codons. In *E. coli*, IRs with cruciform-forming potential are actively placed or excluded in the regulatory regions for the initiation and termination of transcription and translation, indicating their deep involvement or influence in these processes.

**Electronic supplementary material:**

The online version of this article (10.1007/s00294-018-0815-y) contains supplementary material, which is available to authorized users.

## Introduction

“B-form” DNA is the basis of the Watson–Crick model of DNA, and most DNA adopts this structure in vivo. However, several “non-B” DNA structures, including left-handed Z-DNA, cruciforms, triplexes and G-quadruplexes, also exist and are formed with special sequence characteristics or defined symmetry elements. Although numerous attempts have been made to clarify their biological roles, only a few studies have been successful (e.g., McAllister and Achberger 1989; Sinden 1994; Liu et al. 2001; Ohyama 2005; Sumida et al. 2006; Kamiya et al. 2007; Wang and Vasquez 2014; Kanoh et al. 2015). The purpose of the current study is to explore the function of IR sequences with cruciform-forming potential. Genomes contain many cruciform-formable IRs; however, despite extensive research, the “in vivo functions” of these IRs remain enigmatic, as in the cases of other unusual DNA structures. Genome-wide analyses can provide powerful clues toward clarifying the reasons for the existence of IRs with cruciform-forming potential. However, such studies have been limited, and the conclusions almost stay at the stage reporting the regional abundance of these IRs (Lillo et al. 2002; Ladoukakis and Eyre-Walker 2008; Strawbridge et al. 2010; Du et al. 2013). To understand the implications of IRs with cruciform-forming potential in biological functions, more detailed analyses are required. The current study elaborated the analytical method, applied it to the *E. coli* genome and provided more detailed and quantitative results.

Cruciforms can be generated from IR sequences. IRs are widely found in the genomes of prokaryotes and eukaryotes (Warburton et al. 2004; Wang and Leung 2009; Strawbridge et al. 2010; Cer et al. 2013; Du et al. 2013), but not all IRs can form cruciforms. The negative supercoiling of DNA can induce the transition of some of IRs into cruciforms (Lilley 1980; Lilley and Markham 1983; Courey and Wang 1988; Paleček 1991; Van Holde and Zlatanova 1994; Shlyakhtenko et al. 1998; Krasilnikov et al. 1999; Oussatcheva et al. 2004; Kouzine and Levens 2007). In vivo, the dynamic processes of DNA replication and transcription, which generate local underwinding and overwinding of DNA molecules, are the major causes for the generation of negative supercoils (Wu et al. 1988; Schvartzman and Stasiak 2004; Kouzine et al. 2013; Lal et al. 2016). In cruciforms, the minimum length of the repeat unit sequence is generally 6–7 bp, but sometimes it is as short as 5 bp (Sheflin and Kowalski 1985; Iacono-Connors and Kowalski 1986; Müller and Wilson 1987; McMurray et al. 1991; Dai et al. 1997; Dai and Rothman-Denes 1998; Jagelská et al. 2010; Nuñez et al. 2015), and the typical number of nucleotides in a loop ranges from 3 to 6 (Hilbers et al. 1985; Furlong and Lilley 1986; Gough et al. 1986; Nag and Petes 1991; Sinden 1994; Potaman and Sinden 2005). However, even IRs with no spacer can form loops in the resulting cruciform (Gough et al. 1986; Scholten and Nordheim 1986), and larger loops can also be formed (Furlong and Lilley 1986; Müller and Wilson 1987; Damas et al. 2012). Many reports suggested the functional implications of cruciforms and/or IR sequences in DNA replication (Pearson et al. 1996; Zannis-Hadjopoulos et al. 2008; Brázda et al. 2011), transcription (Dai et al. 1997; Dai and Rothman-Denes 1998; Jagelská et al. 2010; Brázda et al. 2012; Coufal et al. 2013), recombination (Lin et al. 1997; Shlyakhtenko et al. 2000; Lobachev et al. 2002; Wang and Leung 2006), and genome or chromosome instability (Wang and Leung 2006; Inagaki et al. 2013; Javadekar and Raghavan 2015). Furthermore, a recent study showed that short IRs with cruciform-forming potential are hotspots for genome instability in human cancer cells (Lu et al. 2015; Bacolla et al. 2016). However, direct evidence for the in vivo presence of cruciform structures and their in vivo functions has not yet been obtained.

Genome-wide computational analyses have been performed for the distributions of *E. coli* IR sequences (Lillo et al. 2002; Du et al. 2013). Lillo et al. examined the numbers and locations of IRs and reported that in most eubacteria, including *E. coli*, IRs with repeat unit lengths of ≥ 9 are preferentially located near the 3′-end of the stop codons (2002). However, they concluded that only some of these IRs fulfill the model requirements characterizing Rho-independent transcription termination, and suggested that other forms of intrinsic termination may be active (2002). Du et al. screened non-B DNA motifs within the context of the operon structure, and reported the similar result that cruciform motifs were strongly enriched downstream of the 3′-end of the stop codons for genes with Rho-independent and Rho-dependent transcription termination mechanisms (2013). In the former study, the *E. coli* genome was partitioned only by the start and the stop codons; in other words, by the coding and noncoding regions, and no information about the transcription initiation and termination sites was provided. Therefore, this study had intrinsic limitations in terms of clarifying whether IRs are actually implicated in transcription initiation or termination. In the latter study, the analysis was performed for operons (including their transcription start sites, TSSs) and noncoding regions. Thus, based only on the strong enrichment of IRs near the 3′-end of the stop codons, the relation of these IRs to the transcription termination mechanism was somewhat far-fetched. Specifically, although each of these studies provided suggestive results, the positional and structural relationships between the IRs and the mRNA ends (or transcription termination sites) were not addressed, but they are absolutely necessary to obtain a definite conclusion about the participation of IRs in the termination of transcription.

In the current study, we constructed the first *E. coli* genome-wide comprehensive map of IRs with cruciform-forming potential. By introducing the information about the DNA positions corresponding to the mRNA ends (i.e., gene ends) for the first time, we could perform more accurate and quantitative analyses for the biological relevance of the focused IRs than in previous studies. We clarified the enrichment of IRs in the following five regions: the adjacent regions downstream of the stop codons, on and around the gene ends, several tens of bp upstream of the start codons within the 5′-UTR, several tens of bp downstream of the start codons, and promoter regions. For the first two types of IRs, most were found to be parts of the intrinsic terminators. In contrast, fewer IRs were present in the small region preceding the start codons.

## Materials and methods

### Genome sequence and gene annotation

The full genome sequence of *E. coli* K-12 MG1655 (U00096.2) was obtained from the NCBI database (http://www.ncbi.nlm.nih.gov). Gene annotations for *E. coli* were from the NCBI database and Conway et al. (2014).

### Partitioning of the genome

The current study focused on protein-coding genes and their flanking regions. Therefore, in the case where two protein-coding genes contain a tRNA gene or genes, a rRNA gene or genes, or a pseudogene or pseudogenes in between, the entire region between the two protein-coding genes was not subjected to the population analyses. ‘Genic’ and ‘intergenic’ regions were defined as follows: genic: ORF (open reading frame), 5′- and 3′-UTRs and OUR-1, -2, and -3 (OUR: overlapping untranslated region; OUR-1, the 5′-UTR of one gene partially or completely overlaps that of another gene; OUR-2, the 3′-UTR of one gene partially or completely overlaps the 5′-UTR of another gene; OUR-3, the 3′-UTR of one gene partially or completely overlaps that of another gene); intergenic: ‘TAN’ (between tandem genes), ‘DIV’ (between divergent genes) and ‘CON’ (between convergent genes). The information about the TSSs and the gene ends for protein coding genes was obtained from Conway et al. (2014) and that about the start codons and the stop codons was obtained from the NCBI database. The terms ‘tandem’, ‘divergent’ and ‘convergent’ refer to the directions of transcription for the abutting genes. For intergenic regions, only those that had two clear ends, such as two TSSs, a gene end and a TSS, or two gene ends, were analyzed.

### IR identifier

We developed the computer program, ‘CIRI’, which identifies the cruciform-formable IRs in a DNA sequence. The CIRI judges a given sequence as a target IR when the repeat unit length is longer than or equal to 5 bp, the spacer length is 0–8 bp and the entire IR length is longer than or equal to 13 bp. The CIRI program was run against the genome sequence of *E. coli*. The CIRI program can be downloaded from our website (http://www.waseda.jp/sem-ohyama/CIRI).

### Genome-wide distribution map of IR sites

Based on the position of the central base pair, the genomic location of each IR site was mapped. When an IR has an even number of base pairs, the central base pair was defined as that immediately downstream of the center position. When an IR is located inside a larger IR, only the outer IR was used for the analyses. The Circos software was used to construct the genome-wide distribution map of the IR sites (Krzywinski et al. 2009). Additionally, we developed a web-based server, ‘Cruciform-formable IRs in the *Escherichia coli* genome (CFIRs-Ec)’ (http://www.waseda.jp/sem-ohyama/CFIRs-Ec), which is an application for browsing the map interactively.

### Regional distribution profiles of IRs

The IR sites were assigned to each partitioned region and mapped. For this purpose, we wrote two homemade scripts. One could sort the IRs found in the *E. coli* genome into the patitioned regions (5′-UTR, ORF, etc.) described above. The other could measure the distance between a given IR and each end of the relevant region. Using these scripts, the regional distribution profiles of IRs were drawn.

### Randomized control sequences and statistical analysis

The *E. coli* genome was partitioned into coding (ORF) and noncoding (non ORF) regions, according to its NCBI database annotations. The sequence randomization was performed by the method of Strawbridge et al. (2010). Briefly, the nucleotides in the coding regions were collected together and then distributed randomly within all of the coding regions of the genome. The same method was also applied to the noncoding regions. This generated a genome in which the coding and noncoding regions were separately shuffled in aggregate, while preserving the positions and lengths of these regions. The resulting randomized genomes were used as “control genomes”. Using 50 randomized genomes, we obtained control data, which were subjected to the statistical analyses. Briefly, using the test datum and the corresponding 50 control data for each bin of 5 bp, the Grubbs test was performed to examine whether the former was a significant outlier.

### Sorting of IRs in and around 3′-UTRs

The TAN samples were aligned according to the distance between the stop codon and the gene end of the upstream-side genes and each region from − 50 to + 500 relative to the third nucleotide of the stop codon of the upstream-side gene was analyzed for IR distribution. The intrinsic terminators were screened using the TransTermHP software (Kingsford et al. 2007) for the screening of the IRs that are parts of the intrinsic terminators.

## Results

### Genome-wide screening of IR sequences with cruciform-forming potential and characteristics of their distributions

We focused on the IRs with a repeat unit length greater than or equal to 5 bp, a spacer length between 0 and 8 bp and an entire IR length is longer than or equal to 13 bp (Supplementary Table S1), which are thought to have the potential to form cruciforms. We did not screen imperfect IRs, because they occur less frequently than perfect IRs (they seem to undergo spontaneous mutations to form more perfect inverted repeats) and require higher energies for cruciform formation (Van Noort et al. 2003). The IRs are named and grouped in the following manner; e.g., R7S5 (the IR with repeat unit length of 7 bp and spacer length of 5 bp), for convenience.

**Fig. 1:**
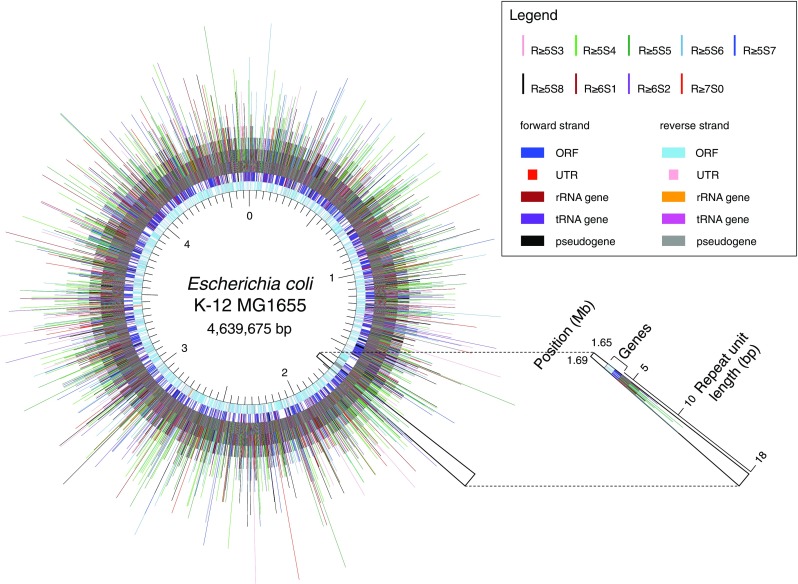
Distribution of IRs in the *E. coli* genome. The position coordinates of the R ≥ 5S ≤ 8 IRs are overlaid on the map of genes with annotation, with their repeat unit lengths shown as line heights. The map can also be browsed interactively in the CFIRs-Ec (http://www.waseda.jp/sem-ohyama/CFIRs-Ec)

**Fig. 2:**
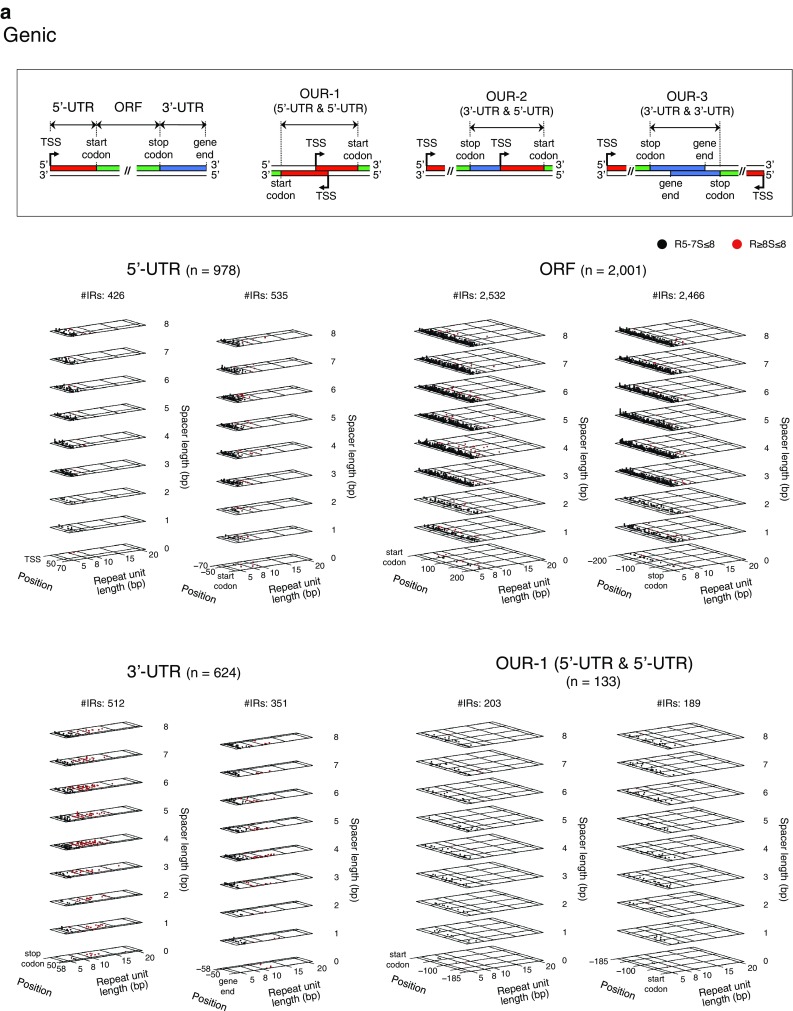

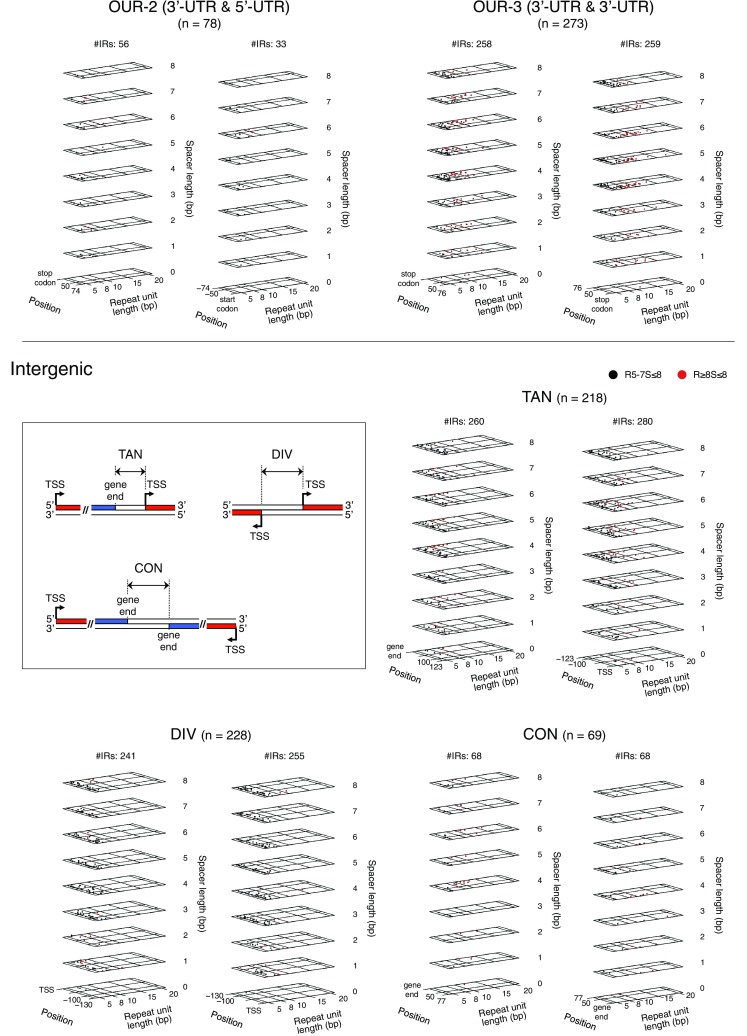

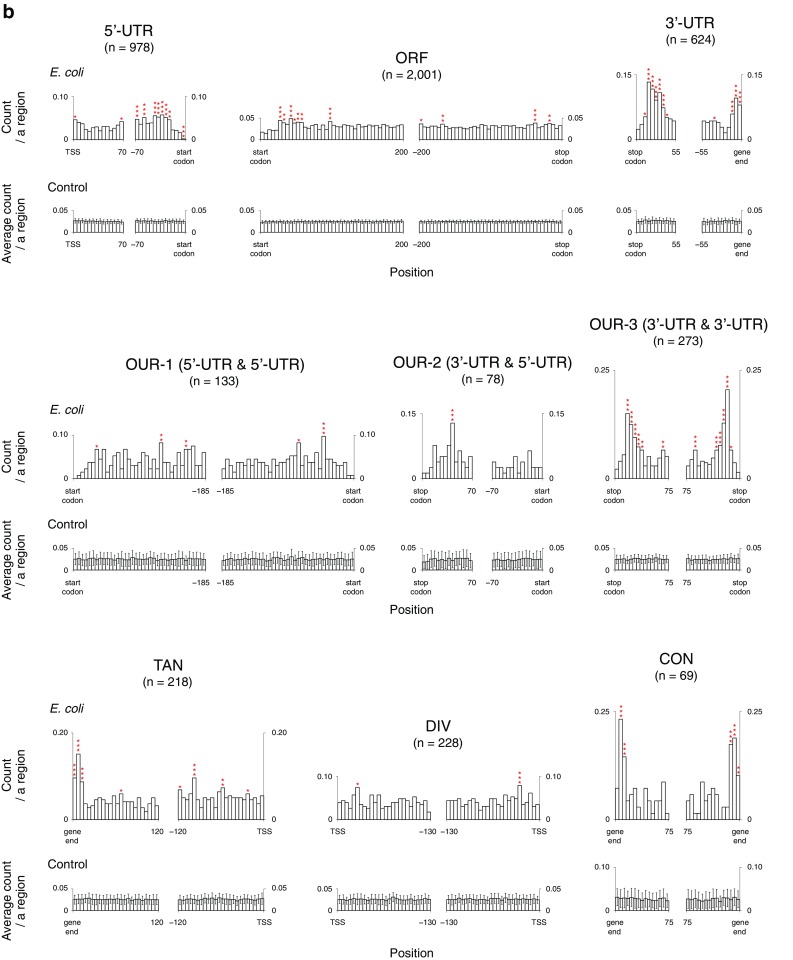
Regional distribution profiles of the R ≥ 5S ≤ 8 IRs. **a** Position of each individual IR. Genic regions are subdivided into ORFs, 5′- and 3′-UTRs, and OUR-1s, -2s, and -3s, as schematically shown at the top. The gene end is defined as the DNA position corresponding to the end of the mRNA. The start codon-coding site and the stop codon-coding site are, respectively, referred to as start codon and stop codon. Intergenic regions are subdivided into TANs, DIVs, and CONs. The IRs are sorted based on the center position. In each data panel, the relationship between the primary structures of the IRs and their positions in the focused region is shown. Position 0 indicates the TSS, the first nucleotide of the start codon, the third nucleotide of the stop codon, or gene end position. The region sizes shown are the averages, except for the ORF panel (Supplementary Table S2). Since the average size of the ORFs is quite large (951 bp), in this case, only the IRs found in the region spanning from the start codon to 200 bp downstream or that from the stop codon to 200 bp upstream are shown. **b** Population histogram of the IRs for each region. Based on the data shown in (**a**), the region-based population histograms of the R ≥ 5S ≤ 8 IRs were generated. The bin size is 5 bp (top). The control data (bottom) were obtained using 50 control genomes (“Materials and methods”). Statistical significance levels were calculated based on the Grubbs test. *P < 0.05, **P < 0.01, ***P < 0.001

At first, we performed a genome-wide analysis for the distribution of the IRs described above, and constructed a comprehensive map for these IRs with the following information: their positions and structures, genes with annotations, and positions of TSSs, gene ends and intrinsic terminators (Fig. 1, http://www.waseda.jp/sem-ohyama/CFIRs-Ec). The map showed that the *E. coli* genome is rich in IRs with the R5S ≤ 8 structure but poor in those with the R ≥ 13S ≤ 8 structure. Subsequently, we examined whether any regional characteristics are associated with the IR occurrence. For this analysis, the genome was divided into the regions shown in Fig. 2a. The gene end was defined as the DNA position corresponding to the end of the mRNA. Based on this definition, the 3′-UTRs were determined. Furthermore, we defined the OUR-1, -2, and -3 regions and they were treated as genic regions. The intergenic regions were classified into TANs, DIVs, and CONs. This analysis excluded the loci of pseudogenes and rRNA and tRNA genes, though these are shown in the map (Fig. 1, http://www.waseda.jp/sem-ohyama/CFIRs-Ec). This is because of the following reasons. For pseudogenes (~ 180 in total), most of them are incomplete for the information on the TSS and the gene end. For rRNA and tRNA genes, it is evident that most of the IRs detected in these loci simply corresponds to the stem and loop structures in these RNA molecules. The magnified map for the distribution of the R ≥ 5S ≤ 8 IRs in these loci is shown in Supplementary Fig. S1.

We found regional characteristics for the IR occurrences, as shown in Fig. 2. Comparatively few R ≥ 8S ≤ 8 IRs are present in ORFs, while they are relatively abundant in 3′-UTRs, OUR-3s, TANs, and CONs (Fig. 2a). In the 3′-UTRs, the R ≥ 8S ≤ 8 IRs frequently occur with their centers positioned  ~ 25 bp downstream of respective stop codons or in the upstream regions close to the vicinity of the gene ends. The frequent occurrence of the IRs in the regions slightly downstream of the stop codons was also found in the OUR-3 panel, as well as in the close vicinity of the gene ends in the TAN and the CON panels, but the loci were in the downstream regions of the gene ends. In contrast, as seen in the 5′-UTR and OUR-1 panels, a small region preceding the start codon does not usually favor the occurrence of IR. Subsequently, we examined whether these findings are statistically significant as compared with the randomized sequences. In the analysis, 50 randomized sequences were generated for each of the coding and noncoding regions (“Materials and methods”). These controls confirmed that most of the above observations are statistically significant, as shown in Fig. 2b (statistically significant regions for the IR enrichment or deficiency are indicated with asterisks). The IR deficiency in a small region preceding the start codon was also confirmed in the 5′-UTR panel, but not in the OUR-1 panel. The latter result might be partly due to the poor statistical power arising from the small number of samples. Regardless of the hypothesis, the much lower occurrence of the IRs in this region was evident even in the OUR-1 panel, as compared to the flanking regions. Importantly, the analysis also clarified that the focused IRs are statistically rich in the regions ~ 20 to ~ 45 bp upstream of the start codons within the 5′-UTR (5′-UTR panel), the regions ~ 25 to ~ 60 bp downstream of the start codons (ORF panel) and promoter regions (TAN and DIV panels).

The stem-loop structures formed in the 3′-end region of the mRNA function in the Rho-independent transcription termination mechanism in *E. coli* (Santangelo and Artsimovitch 2011). In this mechanism, transcription terminates downstream of the IR sequences. Thus, the IR located in the upstream region of the gene end (the position corresponding to the transcript end) may be implicated in this mechanism. However, when a given IR is centered in the downstream region of the gene end, it superficially does not seem to be related to the Rho-independent transcription termination. Nevertheless, such IRs were also frequently found (the TAN and CON panels). The information on the mutual positional relationship among the stop codon, the gene end, and one IR or more IRs in each gene may provide a clue to understanding this result. Clarification can be obtained by scrutinizing the gene structure in and around the 3′-UTR. For this purpose, we examined the structures of the genes that provided the data in the TAN panel in Fig. 2.

**Fig. 3:**
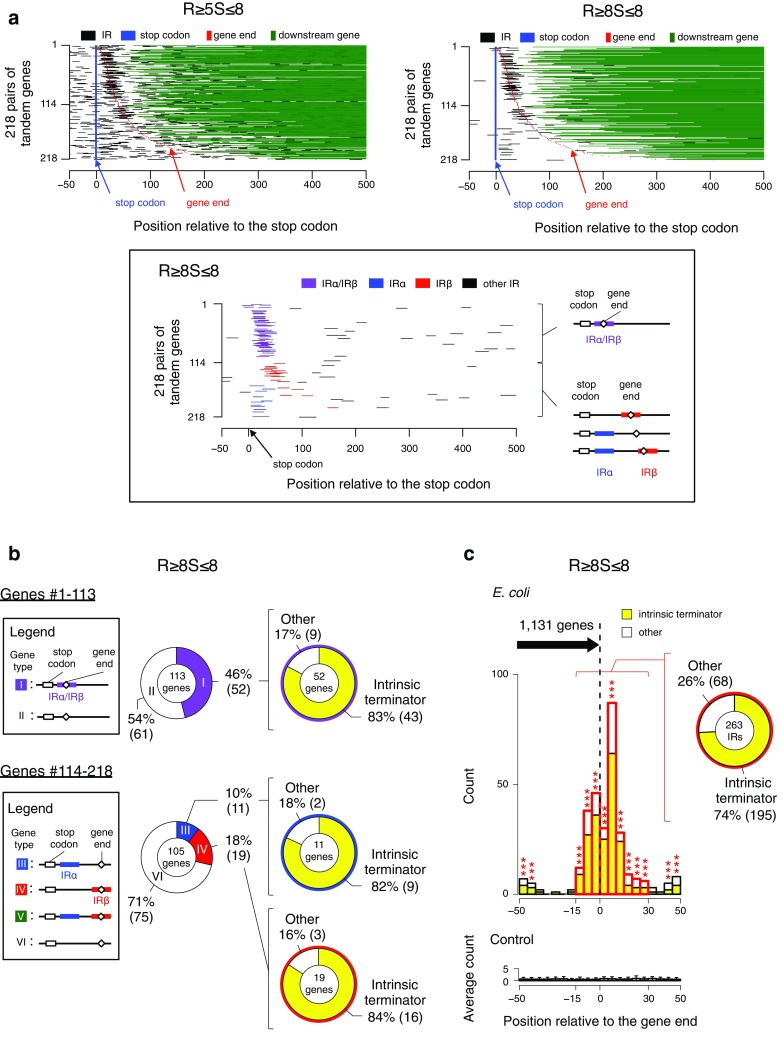
Distribution of the IRs in the gene end region. **a** Distributions of the R ≥ 5S ≤ 8 IRs and the R ≥ 8S ≤ 8 IRs. In total, 218 pairs of the TAN sample genes were used in the analysis. The paired genes are aligned according to the distance between the stop codon (dark blue) and the gene end (red) of the upstream-side genes (the distance gradually increases from top to bottom). Position 0 indicates the third nucleotide of the stop codon. The black lines indicate the IRs. The inset diagrammatically shows the definitions of IRα and IRβ, using the colored lines (see text for details). **b** IR-position-based assortment of genes and occupancies of the intrinsic terminators. According to the presence or absence of the IR, or the position of the IR or IRs, the upstream-side genes were sorted into several types as shown in the insets on the left: for #1–113 genes in (a), types I and II; for #114–218 genes, types III–VI. The two pie charts in the middle show the occupancies of the type I and II genes in the #1-113 genes and those of the type III, IV, and VI genes in the #114–218 genes (the type V gene was not found), respectively. The three pie charts at the right show the occupancies of the intrinsic terminators in the IRs of the type I, III, and IV genes. **c** Population histogram of the R ≥ 8S ≤ 8 IRs for the region centered at the gene end. The 1,131 genes whose end regions (− 50 to + 50 relative to the respective ends) are not invaded by the gene ends of downstream genes were subjected to the analysis. The bin size is 5 bp (top). The control data (bottom) were obtained using 50 control genomes (“Materials and methods”). Statistical significance levels were calculated based on the Grubbs test. ***P < 0.001

In Fig. 3a, 218 pairs of tandem genes (the TAN samples) are aligned according to the distance between the stop codon and the gene end of the upstream-side gene. Three interesting features were found among the upstream-side genes. One is that a clear contrast is seen between the upstream and downstream regions of the stop codons for the occurrence of the R ≥ 8S ≤ 8 IR (right profile): these IRs are scarce in the upstream region of the stop codon, while they are frequently found in the downstream region of the stop codon. Another is that two noteworthy IR distributions exist among the upstream-side genes of nos. 114–218, which are clearly found in the right panel for the R ≥ 8S ≤ 8 occurrence: one population occurs in the adjacent regions downstream of the stop codons, and another population occurs on and around the gene ends. The former and latter IRs are, respectively, referred to as IRαs and IRβs hereafter. The last feature is that the difference in the profiles between R ≥ 5S ≤ 8 and R ≥ 8S ≤ 8 suggests that the IRs with repeat unit lengths of 5–7 bp occur quite randomly in the focused region.

Based on the positions of the R ≥ 8S ≤ 8 IRs, the upstream-side genes of nos. 114–218 can be grouped into several types, as shown in Fig. 3b. The type III genes, each carrying an IRα, and the type IV genes, each carrying an IRβ, were found to account for 10% (11 genes) and 18% (19 genes) of the total of 105 genes, respectively. However, the type V genes that have both IRα and IRβ were not found. Most of these IRs were parts of the intrinsic terminators: the IRαs of 9 genes account for 82% in the IRα-containing 11 genes, and the IRβs of 16 genes account for 84% in the IRβ-containing 19 genes. It was also found that IRαs and IRβs are indistinguishable in the sequence characteristics (data not shown). Although for the upstream-side genes of nos. 1-113, IRα and IRβ could not be separated, most of these IRs (43 genes among the 52 genes; 83%) were also verified to be parts of intrinsic terminators. Finally, using the genes whose end regions (− 50 to + 50 relative to the respective ends) are not invaded by the gene ends of downstream genes, we also examined the occurrence frequency of the R ≥ 8S ≤ 8 IRs there and the occupancy of the intrinsic terminator-composing R ≥ 8S ≤ 8 IRs within them (Fig. 3c). This analysis clarified that the R ≥ 8S ≤ 8 IRs are obviously concentrated around the gene ends, and this phenomenon was statistically significant as compared with the randomized sequences. Importantly, most of them (74%) were parts of the intrinsic terminators.

## Discussion

We constructed the first *E. coli* genome-wide comprehensive map of IRs with cruciform-forming potential. Based on the map, we obtained substantial and quantitative data that allowed us to deeply examine whether these IRs are actually implicated in some genetic processes. Here, based on the current results and those reported previously, we discuss the biological implications of the focused IRs.

### What is renewed by the methodological improvements

Besides the current study, there were two reports with purposes that partially overlapped with the current study (Lillo et al. 2002; Du et al. 2013). At first, we will summarize the differences in the target sequences among the three studies. The targets were as follows: Lillo et al., IRs with a repeat unit length of 4–20 bp and a spacer length of 3–10 bp; Du et al., IRs with a repeat unit length of ≥ 9 bp and a spacer length of 1–10 bp; the current study, IRs with a repeat unit length of ≥ 5 bp, a spacer length of ≤ 8 bp and a total length of ≥ 13 bp (Supplementary Table S1). Our screen and that by Du et al. targeted cruciform-formable sequences in the genome, while Lillo et al. apparently did not specifically target them. Considering that the shortest stem in a putative in vivo originated cruciform was 5 bp (Sheflin and Kowalski 1985; Iacono-Connors and Kowalski 1986; Dai et al. 1997; Dai and Rothman-Denes 1998), Lillo et al. screened an excess of IRs from the viewpoint of cruciform-forming potential. In contrast, Du et al. missed the IRs with repeat unit lengths of 5–8 bp, which are frequently found in the genome.

The biggest difference among the three studies is the map construction: the current study constructed the first genome-wide comprehensive map of IRs with cruciform-forming potential, which includes the detailed positional and structural information of these IRs, as well as information about genes with annotations, TSSs, gene ends and intrinsic terminators (Fig. 1, http://www.waseda.jp/sem-ohyama/CFIRs-Ec). In contrast, the two preceding studies did not provide any maps. Although a web-based server for detecting palindromes has recently become available (Brázda et al. 2016), it does not provide a comprehensive map. Another notable methodological difference is the genome partitioning. The current study was the first to include the information about the gene ends, corresponding to the mRNA ends, in the analysis. This allowed us to exclude the contamination of IRs belonging to neighboring genes (Fig. 3c). More importantly, these three major methodological differences allowed us to draw correct conclusions and clear up the ambiguities in the preceding studies, which are summarized as follows.

Lillo et al. reported the abundance of IRs with a repeat unit length of ≥ 9 bp in the regions downstream of the stop codon (2002), while we detected the enrichment of IRs with a repeat unit length of ≥ 8 bp in the corresponding region. Thus, the findings seem to be roughly similar to each other. However, regarding the relevance of these IRs to intrinsic terminators, the two studies led to quite different conclusions. Lillo et al. concluded that only some of the observed IRs satisfy the model requirements characterizing Rho-independent transcription termination. In contrast, using the genes with end regions (− 50 to + 50 relative to the respective ends) that are not invaded by the gene ends of downstream genes, we clarified that the R ≥ 8S ≤ 8 IRs are concentrated in the region spanning from the stop codon to the gene end region, and 74% of the R ≥ 8S ≤ 8 IRs located around the gene ends are parts of the intrinsic terminators (Fig. 3c). There are two reasons for this large difference. One is that we introduced the gene end information in the analysis for the first time, which clarified whether the IRs belonged (i.e., could exclude contamination of IRs belonging to neighboring genes that may have some other mechanistic role) and, as a result, improved the accuracy of the analysis. The other is that the capability of the computer program to correctly identify terminator sequences has improved, as compared to the analysis in 2002.

Du et al. reported the enrichment of cruciform motifs (designation in their study) in the promoter regions of operons, especially near TFBSs (transcription factor binding sites), and downstream of operon ends (2013). In the current study, the regions enriched in IRs with cruciform-forming potential, as compared to the randomized control sequences, were the adjacent regions downstream of the stop codons, on and around the gene ends, ~ 20 to ~ 45 bp upstream of the start codons within the 5′-UTR, ~ 25 to ~ 60 bp downstream of the start codons, and promoter regions. Furthermore, we also found an IR deficient region; i.e., the small region preceding the start codons. Thus, the clear differences between the two studies lie in the results for the regions upstream and downstream of the start codon, in which we found the enrichment of the focused IRs while Du et al. did not, and the results for the small region preceding the start codons, for which the IR deficiency was found only in the current study. These differences were probably caused by the difference in the screening targets between the two studies, as described.

Here, we must also note that there was another report in which the occurrence of small IRs was analyzed using only protein coding regions (Ladoukakis and Eyre-Walker 2008). Although this report showed the less frequent occurrence of the focused IRs in the *E. coli* ORFs than in the control randomized sequences, it did not examine the profiles of the dissected ORFs. This is probably why the study failed to find the enrichment of the IRs in the small regions (i.e., ~ 25 to ~ 60 bp downstream of the start codon). Some of the IRs found frequently ~ 20 to ~ 45 bp upstream of the start codons within the 5′-UTR may function as riboregulators for transcription (Millman et al. 2017). Similarly, some occurring in the regions ~ 25 to ~ 60 bp downstream of the start codons may also have the same function. Further studies will be required to determine the specific functions of these two populations.

Another major difference between the report by Du et al. and the current study is regarding the IRs found downstream of the stop codons. By introducing the information on the gene ends, we could obtain precise positional information for each IR relative to the gene end, which allowed us to sort the IRs into several types and provide quantitative data for the first time, as shown in Fig. 3b, c. Finally, we also note a common finding between the two studies, regarding the enrichment of the focused IRs in promoter regions. The populations marked with asterisks in the DIV and TAN (the right side) panels in Fig. 2b may have a TFBS in the close vicinity, as suggested by Du et al.

### Possible reasons why IRs frequently occur in the gene end region and are excluded in the small region preceding the start codon

Due to the lack of data for the mRNA ends, previous studies could not accurately clarify the positions of IRs relative to the gene ends, and thus it was difficult to correctly screen for the IRs that are actually involved in transcription termination. Therefore, a definite conclusion could not be obtained. In the current study, we clarified that the region spanning from the stop codon to the gene end is rich in the R ≥ 8S ≤ 8 IRs (Fig. 2a). Most of them were parts of the intrinsic terminators (Fig. 3). Thus, these IRs seem to be used for Rho-independent termination of transcription. However, to confirm this proposal, we must explain why many R ≥ 8S ≤ 8 IRs stretch over the gene ends. The Rho-independent transcription termination usually occurs several bp downstream from the terminators (Ray-Soni et al. 2016; Porrua et al. 2016). Thus, the IRs “must” always be located in the upstream of the gene ends, if they are used in transcription termination. This contradiction can be explained as follows. Annotating the 3′-ends of bacterial mRNAs is a much more difficult endeavor than mapping the TSSs, because there is currently no method for their enrichment (Creecy and Conway 2015). Accordingly, the transcriptome data that we used (Conway et al. 2014) probably contained the data for the 3′-truncated transcripts, and this seems to be an inevitable problem at present. Finally, there are two points to be discussed in relation to the issue of transcription termination. A small population of the R ≥ 8S ≤ 8 IRs was not part of the intrinsic terminators (Fig. 3c). However, they may also function in transcription termination with the Rho-dependent mechanism, as suggested by Du et al. (2013). The other question is why the distances between the IRα-harboring terminators and the gene ends (transcript ends) are much longer (~ 100 to ~ 400 bp) than the usual cases (Fig. 3a). At present, we lack a plausible explanation for this phenomenon. An unknown relationship may still remain between the intrinsic terminators and the termination points of transcription.

We clarified that the small region preceding the start codon is the IR-deficient region (Fig. 2). Diminishing the probability of forming a stem-loop RNA structure in the small region preceding the start codon in mRNA may be one reason for this phenomenon, because this structure would negatively influence translation initiation. However, another reason may be related to the Shine-Dalgarno (SD) sequence in prokaryotes, which is generally located ~ 8 bases upstream of the start codon (Shine and Dalgarno 1975). This conserved sequence is involved in the process of translation initiation. Accordingly, to reserve the integrity of the SD sequence, IRs may be excluded in the region as the DNA sources that can destroy the integrity. In summary, there seems to be an RNA-based reason for the low IR occurrence in the small region preceding the start codon.

### Function at the DNA level

Regarding the promoter regions of genes, Du et al. reported a preference for cruciform motifs near TFBSs (2013). Although we did not examine the positional relationship between TFBSs and the focused IRs, we also found several regions where the enrichment of the focused IRs was statistically significant, as compared with the randomized control sequences (Fig. 2b, the TAN and DIV panels). These regions may contain TFBSs. However, functions of the IRs are unclear. They may function as the first recognition signal before transcription factors find and bind to their binding sites. Zhabinskaya and Benham reported that the cruciform motifs located in the immediate 5′-flanking regions of *E. coli* genes have a very high melting probability (2013). Therefore, some of the IRs with such localizations may be involved, in part, in the open promoter complex formation, to facilitate transcription initiation. In contrast, other such IRs may be used in transcriptional repression, as in the case reported by Horwitz and Loeb (1988). In their study, a cruciform repressed the transcription of the downstream gene in *E. coli*. Regardless of the hypotheses, these IRs presumably function at the DNA level.

## Conclusion

Using *E. coli*, we constructed the first genome-wide comprehensive map for IRs with cruciform-forming potential, which includes information about not only these IRs but also genes with annotations and positions of TSSs, gene ends and intrinsic terminators. The following five regions are statistically rich in IRs with cruciform-forming potential: the adjacent regions downstream of the stop codons, on and around the gene ends, ~ 20 to ~ 45 bp upstream of the start codons within the 5′-UTR, ~ 25 to ~ 60 bp downstream of the start codons and the promoter regions. The R ≥ 8S ≤ 8 IRs are concentrated in the first two regions, and most of them are parts of the intrinsic terminators. The small region preceding the start codons is the IR-deficient region. Based on these findings, we conclude that IRs with cruciform-forming potential are actively placed or excluded in the regulatory regions of initiation and termination of transcription and translation in *E. coli*, indicating their deep implications and effects in these processes.

## Electronic supplementary material

Below is the link to the electronic supplementary material.

Supplementary material 1 (PDF 389 KB)
