# Correction to: Requirement or exclusion of inverted repeat sequences with cruciform-forming potential in *Escherichia coli* revealed by genome-wide analyses

**DOI:** 10.1007/s00294-018-0881-1

**Published:** 2018-08-22

**Authors:** Osamu Miura, Toshihiro Ogake, Takashi Ohyama

**Affiliations:** 1 Department of Biology, Faculty of Education and Integrated Arts and Sciences, Waseda University, 2-2 Wakamatsu-cho, Shinjuku-ku, Tokyo, 162-8480 Japan; 2 Major in Integrative Bioscience and Biomedical Engineering, Graduate School of Science and Engineering, Waseda University, 2-2 Wakamatsu-cho, Shinjuku-ku, Tokyo, 162-8480 Japan

## Correction to: Current Genetics (2018) 64:945–958 10.1007/s00294-018-0815-y

The article ‘Requirement or exclusion of inverted repeat sequences with cruciform-forming potential in *Escherichia coli* revealed by genome-wide analyses' written by Osamu Miura, Toshihiro Ogake, Takashi Ohyama was originally published online on SpringerLink with open access.

The copyright was incorrect in the article HTML version and it should read as ‘The Author(s) 2018’.

The original article HTML version has been corrected.

