# Supplementary material for: Requirement or exclusion of inverted repeat sequences with cruciform-forming potential in Escherichia coli revealed by genome-wide analyses
Source: Curr Genet. 2018 Feb 27;64(4):945–58. doi: 10.1007/s00294-018-0815-y (PMC6060812; doi:10.1007/s00294-018-0815-y)
Supplement: Supplementary file 1 — Supplementary material 1 (PDF 389 KB) [file 294_2018_815_MOESM1_ESM.pdf]

Requirement or exclusion of inverted repeat sequences with cruciform-forming potential in *Escherichia coli* revealed by genome-wide analyses

Current Genetics

Osamu Miura[1,2], Toshihiro Ogake[2] and Takashi Ohyama[1,2,*]

[1] Department of Biology, Faculty of Education and Integrated Arts and Sciences, Waseda University

[2] Major in Integrative Bioscience and Biomedical Engineering, Graduate School of Science and Engineering, Waseda University

* To whom correspondence should be addressed. Email: ohyama@waseda.jp

Supplementary Table S1

Supplementary Table S1 Comparison of the parameters for detecting the focused IRs in the relevant studies.

Reference	Repeat unit length (bp)	Spacer length (bp)	Entire length (bp)
Lillo et al. 2002	4–20	3–10	11–50
Ladoukakis and Eyre-Walker 2008	6–9	≤50	12–68
Du et al. 2013	≥9	1–10	≥19
Current study	≥5	≤8	≥13

Supplementary Table S2

Supplementary Table S2 The average size of each region.

Region	Count	Average size (bp)
5'-UTR	978	70
ORF	2,001	951
3'-UTR	624	58
OUR-1 (5’-UTR & 5’-UTR)	133	185
OUR-2 (3’-UTR & 5’-UTR)	78	74
OUR-3 (3’-UTR & 3’-UTR)	273	76
TAN	218	123
DIV	228	130
CON	69	77

Supplementary Fig. S1a

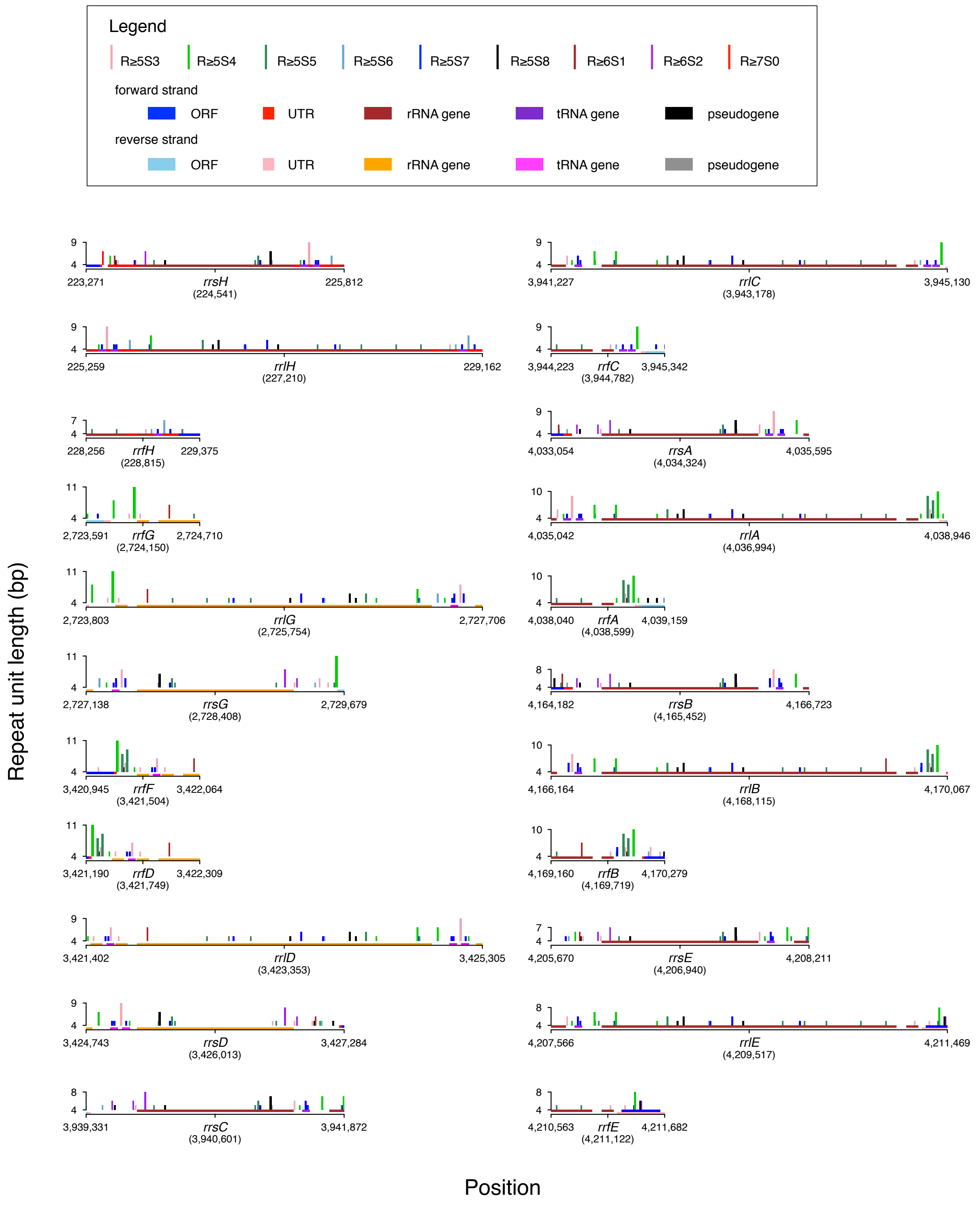

Supplementary Fig. S1 Maps of the distributions of the R≥5S≤8 IRs in rRNA gene loci (a) and tRNA gene loci (b). The genes are shown with 500 bp (rRNA gene) or 50 bp (tRNA gene) flanking regions. The center position of each gene is indicated in parenthesis.

Supplementary Fig. S1b

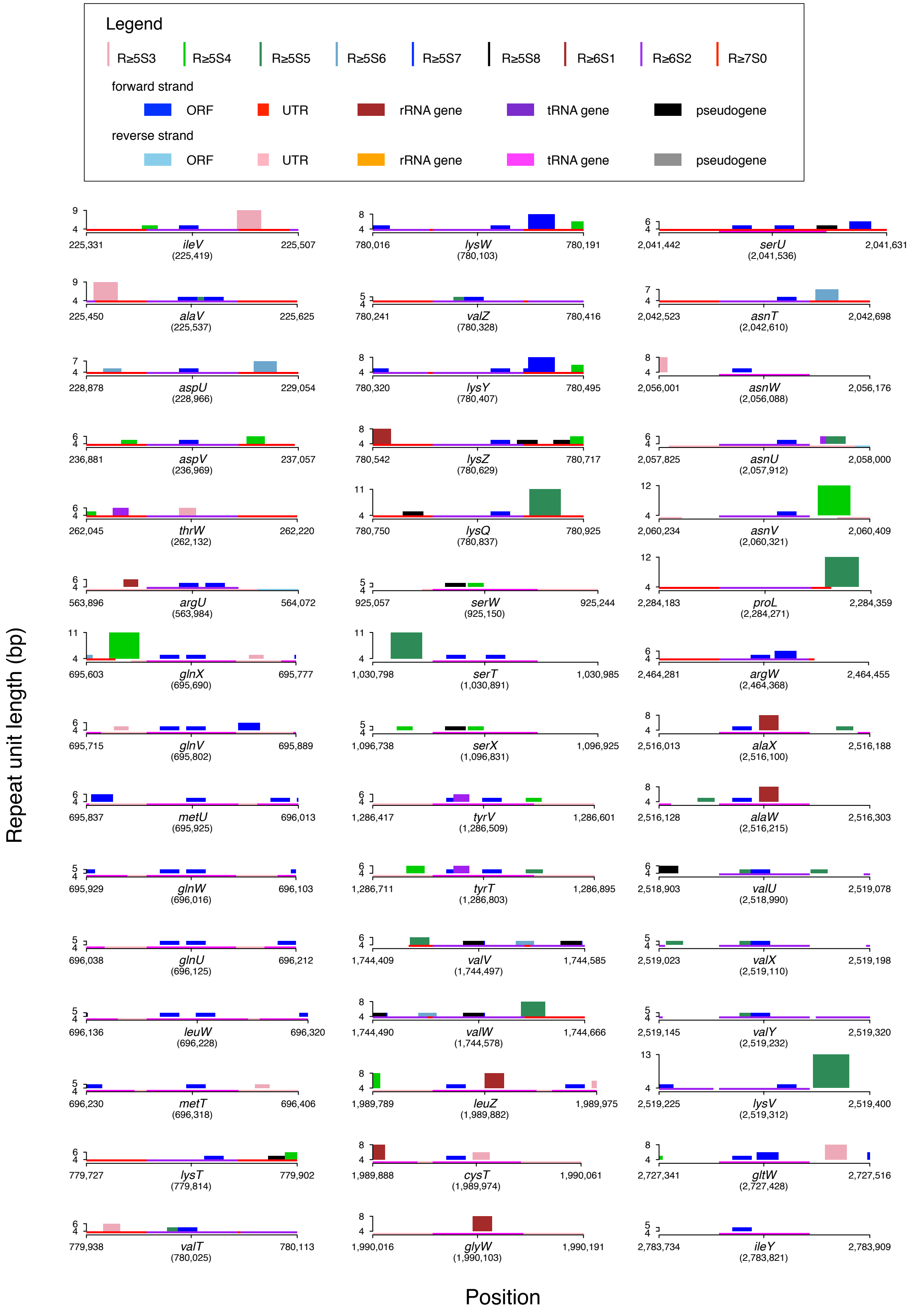

Supplementary Fig. S1 Maps of the distributions of the R≥5S≤8 IRs in rRNA gene loci (a) and tRNA gene loci (b). The genes are shown with 500 bp (rRNA gene) or 50 bp (tRNA gene) flanking regions. The center position of each gene is indicated in parenthesis.

Supplementary Fig. S1b (continued)

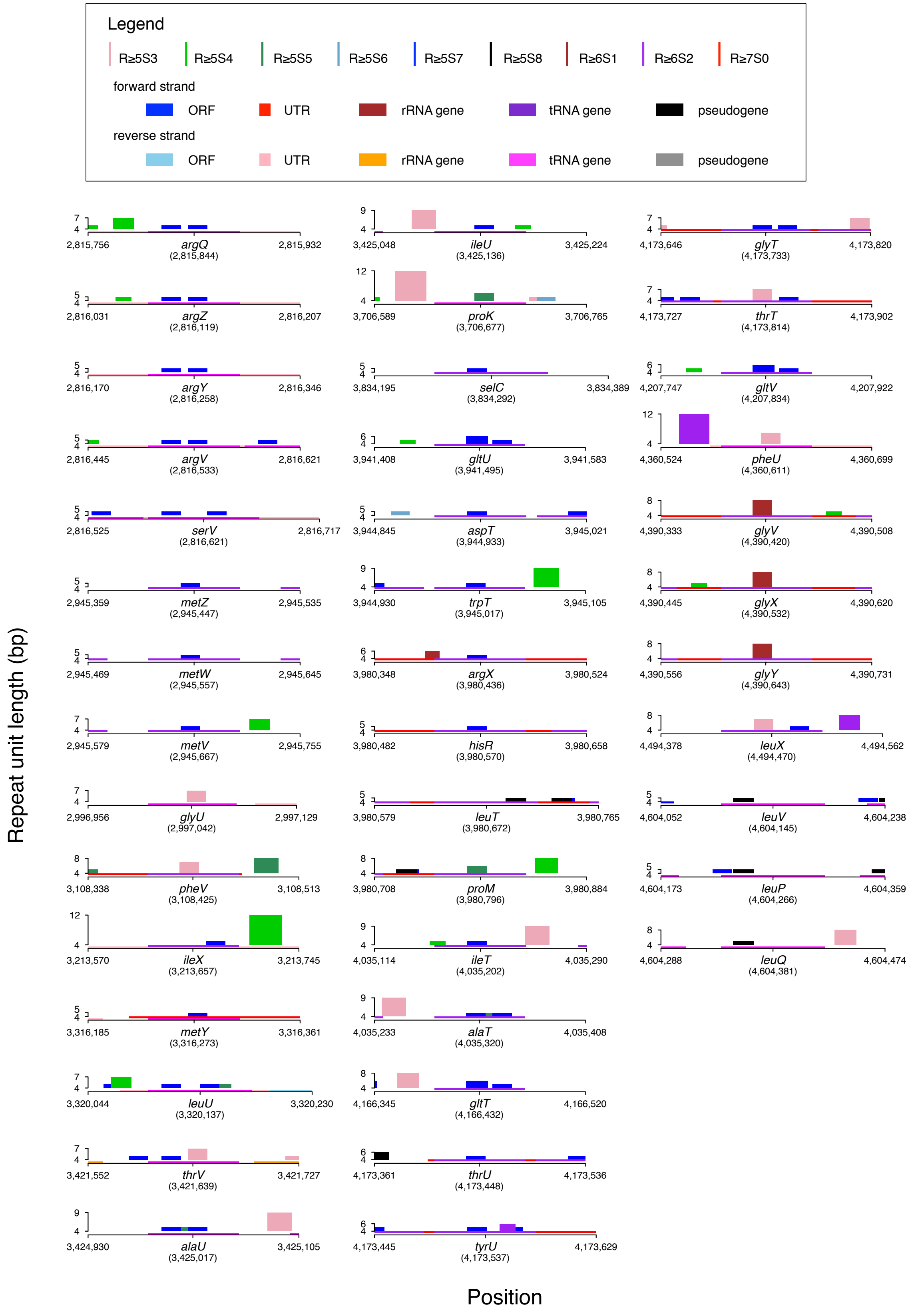

Supplementary Fig. S1 Maps of the distributions of the R≥5S≤8 IRs in rRNA gene loci (a) and tRNA gene loci (b). The genes are shown with 500 bp (rRNA gene) or 50 bp (tRNA gene) flanking regions. The center position of each gene is indicated in parenthesis.
